# Bioengineering Methods of Analysis and Medical Devices: A Current Trends and State of the Art

**DOI:** 10.3390/ma13030797

**Published:** 2020-02-10

**Authors:** Marco Cicciù

**Affiliations:** Department of Biomedical and Dental Sciences and Morphological and Functional Imaging, Messina University, 98122 Messina, Italy; mcicciu@unime.it

**Keywords:** biomedical, bioengineering, methods, finite element analysis, von mises, dentistry, biotechnologies, biomaterials, materials

## Abstract

Implantology, prosthodontics, and orthodontics in all their variants, are medical and rehabilitative medical fields that have greatly benefited from bioengineering devices of investigation to improve the predictability of clinical rehabilitations. The finite element method involves the simulation of mechanical forces from an environment with infinite elements, to a simulation with finite elements. This editorial aims to point out all the progress made in the field of bioengineering and medicine. Instrumental investigations, such as finite element method (FEM), are an excellent tool that allows the evaluation of anatomical structures and any facilities for rehabilitation before moving on to experimentation on animals, so as to have mechanical characteristics and satisfactory load cycle testing. FEM analysis contributes substantially to the development of new technologies and new materials in the biomedical field. Thanks to the 3D technology and to the reconstructions of both the anatomical structures and eventually the alloplastic structures used in the rehabilitations it is possible to consider all the mechanical characteristics, so that they could be analyzed in detail and improved where necessary.

In recent years, many advances have been made in the field of bioengineering and biotechnology. Many methods have been proposed for the in-silica study of anatomical structures and alloplastic structures. Some of these methods involve the use of computerized simulations, without, therefore, being invasive towards patients and being able to evaluate which are the best therapeutic choices only by processing data. A document of the Federal Drug Administration (FDA) issued on September 2016 stated how “Computational modeling and simulation studies, together with bench, nonclinical *in vivo*, and clinical studies, can be used to evaluate the safety and effectiveness of medical devices”. Finite element method represents a possibility of recreating real condition simulating the stress of static device placed in a dynamic body or structure (Figure 1, Figure 2 and Figure 3) [1,2,3,4,5,6,7,8,9]. Dentistry, and therefore, implantology, prosthetics, implant prosthetics, or orthodontics in all their variants, are medical and rehabilitative branches that have benefited greatly from these methods of investigation to improve the predictability of rehabilitations [10]. The finite element method (FEM) and finite element analysis (FEA) are valid methods in biomedical field and could help to develop new technologies [11,12,13]. This method involves the simulation of mechanical forces from an environment with infinite elements, the real one, to a simulation with finite elements. Internal stresses and strains could therefore be obtained, which are essential, and whose direct measurement would be difficult if an adequate computational model was not available. Even the finite element method is in fact considered a valid tool in the experimental approach in order to predict the distribution of loads between the different structures at both the whole body and single organ level by static and/or dynamic analysis as it allows for modeling irregular geometry structures made up of materials with complex properties and easily simulating situations of difficult loads and boundary conditions [14,15,16,17,18,19,20]. 

Parametric analysis is exploited in different fields of dentistry. In some studies, FEA analysis is used to evaluate orthognathic surgery healing phases and results. It shows the impact of maxillofacial surgery in 3D. This type of analysis could be easily performed before treatment or surgery for stress and strain distribution or for load evaluation [21,22,23]. FEM analysis could be used to investigate dentin loading condition, evaluating the anisotropy, and the elastic properties of this tissue. FEA could be used to investigate how an increased implant diameter reduce stress on surrounding tissue, especially under oblique loading on fixtures. A further role in making FEM evaluations, before the interventions and in a short time, is given by the fact that they could now obtain reliable images of the oral structures, thanks to the digital impression techniques that are increasingly common [24,25]. Crown FEM analysis demonstrate how there is no ideal material and reflected different features on different load. “The zirconia offers the advantages of high aesthetic but the low resistance of fracture on long-term” [26].

Having a high-resolution CT radiology examination, 3D geometry of a specific implant, and 3D reconstruction of the patient-specific anatomy of the implant site, it is possible, for example, to calculate the peri-implant stress during the loads induced by mastication [27]. It is possible to simulate surgical procedures with different types of implants and to verify how (theoretically) the medullary bone responds to the stresses induced by the surgical procedure and the osseointegration of the implant itself. This makes it possible to evaluate the system that is theoretically more suitable for that specific clinical case and still in orthodontics [28,29]. These are the most concrete and realistic applications of this technique. In fact, it is possible to simulate physically correct dental movements based on known forces [1,30,31,32,33,34]. This allows for checking, for example, the best positioning of an orthodontic element by evaluating the various positions and the implications that derive from it.

We are proud that this topic has been largely treated in this Special Issue (SI). The topic widely treated in this SI aims to evaluate the survival and success rates of dental implant-supported prosthesis. The clinical success depends on several factors, including physical and chemical properties of implant materials, such as microstructure, its surface composition and characteristics, as well as design factors. For this reason, the interaction between the medical and engineering fields is a fundamental step in order to produce predictable devices, tried in vitro and then applied in vivo on patients. 

At this moment the seven papers have been published in the special issue and it is possible to find them at the following link: https://www.mdpi.com/journal/materials/special_issues/dental_implants_materials.

The published papers covered different aspects about chemical and physical features of dental implants as well as the effectiveness of new tools for guide surgery in dental implants positioning, while also treating the clinical aspects related to the bone response and the aesthetic value of the oral surgery related to this kind of treatment.

Due to the high impact factor of “Materials MDPI” we encourage further submission of new manuscripts covering the SI theme. The published researches will have great visibility and rapid diffusion related to high value of the Journal in the International Research Committee.

## Figures and Tables

**Figure 1:**
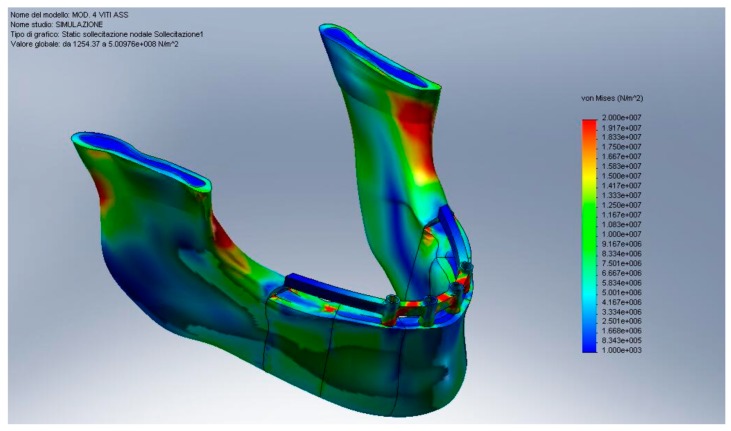
Mandibular von Mises analysis with all on four implant prosthetic rehabilitation.

**Figure 2:**
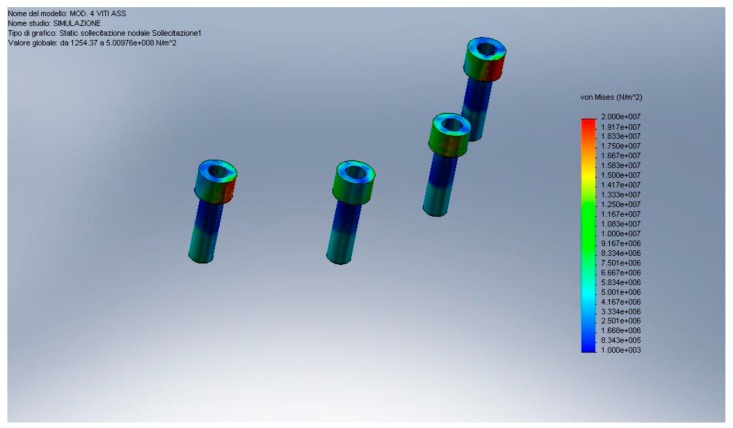
Passant abutment screws von Mises analysis.

**Figure 3:**
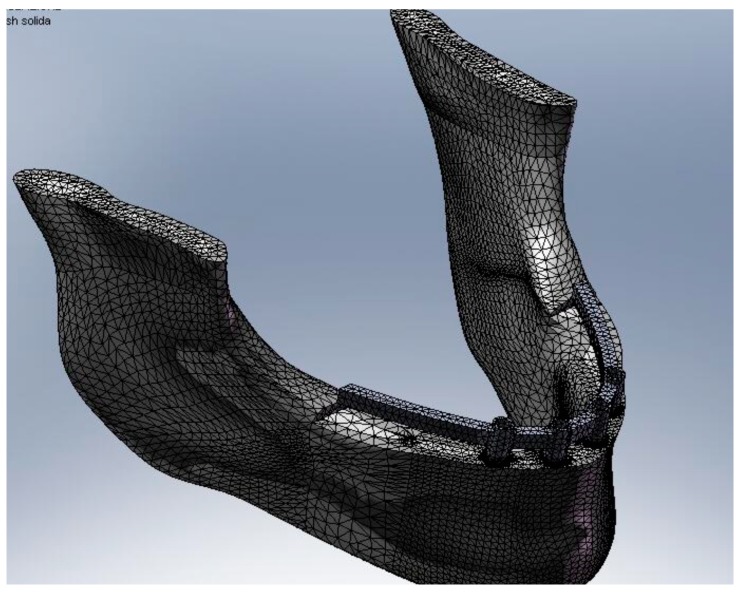
Mandibular finite element method (FEM) model.
